# Sustainability in Quality Improvement (SusQI): challenges and strategies for translating undergraduate learning into clinical practice

**DOI:** 10.1186/s12909-021-02963-7

**Published:** 2021-10-30

**Authors:** Oliver Marsden, Philippa Clery, Stuart D’Arch Smith, Kathleen Leedham-Green

**Affiliations:** 1 University of Bristol Medical School, Bristol, UK; 2 University Hospitals Bristol NHS Foundation Trust, Bristol, UK; 3 Centre for Sustainable Healthcare, Oxford, UK; 4 Medical Education Research Unit, Imperial College London, London, UK

**Keywords:** Medical education, Quality improvement, Sustainable healthcare

## Abstract

**Background:**

The healthcare sector is a major contributor to climate change and there are international calls to mitigate environmental degradation through more sustainable forms of clinical care. The UK healthcare sector has committed to net zero carbon by 2040 and sustainable healthcare is a nationally mandated outcome for all UK graduating doctors who must demonstrate their ability to address social, economic, and environmental challenges. Bristol Medical School piloted successful Sustainability in Quality Improvement (SusQI) workshop, but identified challenges translating classroom learning into clinical practice. This paper aims to identify and address those challenges.

**Methods:**

We conducted five focus groups that identified and iteratively explored barriers and facilitators to practice among medical students, comparing a range of experiences to generate a conceptual model. We then combined our findings with behaviour change theory to generate educational recommendations.

**Results:**

Students that applied their learning to the clinical workplace were internally motivated and self-determined but needed time and opportunity to complete projects. Other students were cautious of disrupting established hierarchies and practices or frustrated by institutional inertia. These barriers impacted on their confidence in suggesting or achieving change. A minority saw sustainable healthcare as beyond their professional role.

**Conclusions:**

We present a series of theoretically informed recommendations. These include wider curricular engagement with concepts of sustainable clinical practice; supportive workplace enablement strategies such as workplace champions and co-creation of improvement goals; and time and headspace for students to engage through structured opportunities for credit-bearing project work.

**Supplementary Information:**

The online version contains supplementary material available at 10.1186/s12909-021-02963-7.

## Background

Climate change and environmental degradation have been recognised as both a threat to human health and a catalyst for new and improved practices [1, 2]. One such practice is the introduction of sustainable healthcare into undergraduate medical curricula.

The Sustainable Development Unit of the UK’s National Health Service (NHS) defines sustainable healthcare as “working across the health system and partners to deliver healthcare that delivers on the triple bottom line, i.e., simultaneous financial, social and environmental return on investment. It includes adapting how we deliver services, health promotion, more prevention, corporate social responsibility and developing more sustainable models of care” [3]. NHS England has committed to carbon neutrality by 2040 [4] and the UK’s General Medical Council (GMC) has mandated that by 2020 all graduating doctors “must be able to apply the principles, methods and knowledge of sustainable healthcare to medical practice” and “be able to apply the principles and methods of quality improvement to improve practice” [5]. Quality improvement (QI) and sustainable healthcare have therefore become part of the core curriculum at all UK medical schools, and all UK practising doctors are expected to demonstrate engagement with QI as part of postgraduate training and professional revalidation.

QI and sustainable healthcare have become priority areas for Health Education England [6] which commissioned the Centre for Sustainable Healthcare, in collaboration with the Health Foundation and King’s College London, to combine these two principles into a practice known as Sustainability in Quality Improvement (“SusQI”). SusQI supports practitioners in improving and maintaining patient care through established QI strategies whilst attending to the triple bottom line, thereby creating social, economic, and environmental value [7, 8].

$$\boldsymbol{Sustainable}\kern0.5em \boldsymbol{value}\kern0.5em=\frac{\boldsymbol{Outcomes}\kern0.5em \boldsymbol{for}\kern0.5em \boldsymbol{patients}\kern0.5em \boldsymbol{and}\kern0.5em \boldsymbol{populations}}{\begin{array}{*{20}l} \boldsymbol{Environmental}\kern0.5em +\kern0.5em\boldsymbol{social}\kern0.5em +\kern0.5em\boldsymbol{financial}\kern0.5em \boldsymbol{impacts}\\ {}\kern4.5em\left(\boldsymbol{the}\hbox{ }\boldsymbol{triple}\kern0.5em \boldsymbol{bottom}\kern0.5em \boldsymbol{line}\hbox{}\right)\end{array}}$$

Mortimer suggests clinical strategies to reduce the environmental impact of healthcare whilst maintaining the quality of patient care [9]. These are described in the SusQI Educators Pack [10] and include:

- health promotion and disease prevention
- supporting patients in managing their long-term conditions more effectively, efficiently, and sustainably e.g., through education, health coaching, social prescribing, and care planning
- creating lean clinical pathways that are more effective and efficient
- choosing options that are less damaging to the environment such as cleaner inhalers and anaesthesia
- ethical resource allocation, procurement and waste streams.

Strategies to improve the social value of healthcare include attention to equity, social determinants of health, social justice, safety, well-being, advocacy, empowerment, participation and access to healthcare [11]. Considerable co-benefits to sustainable healthcare have been described [12]. For example, lower carbon care is often easier, cheaper over time, and better for patients. Linking sustainability with quality improvement therefore has the potential for a strongly positive net impact on both patient care and service provision.

The SusQI toolkit has been piloted at UK medical schools including Bristol, and this research is part of that pilot. Clery et al. evaluated the Bristol SusQI workshop which was successful in building motivation and self-rated skills, and students described transformational new perspectives on sustainable healthcare [13]. Some students, however, described challenges getting started on SusQI projects in the clinical workplace. This research aims to build explanatory theory from participant experiences so that further implementation of the SusQI toolkit is both evidentially and theoretically informed and the impacts in the clinical workplace are maximised.

## Methods

### Research questions

What enabled some students to engage in SusQI? Where this was not the case, what barriers did students encounter, and what strategies might foster successful engagement?

### Paradigm and approach

We adopted a participatory constructivist approach to research, aiming to create conceptual understanding through an inductive analysis of focus group data, and involving students and teachers as co-creators of knowledge. Our analysis included an abductive inference stage exploring our findings through existing theories of change to identify ‘what might help’.

### Research team & reflexivity

OM is a medical student from the same cohort as participants who conducted the focus groups and initial coding as part of a credit-bearing project. PC and SdAS are clinical academics who facilitated the SusQI workshops. KLG is an educational researcher from another institution. KLG and PC trained and supported OM in data acquisition and analysis methods. All authors contributed to the overarching analysis and write-up.

Participatory inquiry involves teachers, students and co-researchers. It supports ‘from within’ perspectives and insights, however the potential for bias requires mitigation [14]. Peer-facilitated focus groups can reduce self-censure and deference, and enhance understanding of idiosyncratic terms and phrases [15], however, there is the potential for recruitment or affirmation bias based on participants’ preconceptions of the facilitator. We mitigated this by aiming for a neutral tone to focus group recruitment and facilitation and auditing so that all perspectives including negative ones were included in the analysis. Our positionality is that action to reduce environmental degradation is more desirable than inaction.

### Intervention

A SusQI workshop was offered as part of compulsory core teaching to all third-year medical students at Bristol Medical School (*n* = 342). There were 10–24 students in each workshop which took place in January–February 2020. Attendance was estimated at > 80% with non-attenders expected to catch-up via a recording. Students were given pre-reading, and the live workshops involved central teaching telecast to eight clinical sites with locally facilitated workshops. Facilitators used the SusQI educators’ toolkit which is freely available from the Centre for Sustainable Healthcare’s website [10]. Students were presented with the NHS’s environmental impacts which were linked to health outcomes. They explored established QI strategies (goal setting, driver diagrams, stakeholder analysis and process mapping) as well strategies for measuring environmental and social impacts. They watched videos of local clinicians who had completed SusQI projects and discussed ideas for projects in small groups. Students were then paired with a local Clinical Teaching Fellow to support student SusQI projects. Projects were neither compulsory nor summatively assessed, however, a follow-up workshop to discuss progress was planned. A detailed description and evaluation of these workshops is available [13].

### Participants and context

All students that had attended a SusQI workshop were eligible to participate. Students were invited to participate via email and social media. This recruited 17 participants which we divided into 5 online focus groups. Participants were in their third year of a five-year MBChB degree with clinical contact in all years. This was the first formal QI training within their course. Focus groups were conducted 4 months after the workshop to allow time for students to start projects. The completion of projects and follow-up workshops were interrupted by the COVID-19 pandemic.

### Data generation and preparation

The study had ethical approval from the University of Bristol (#98065). Participants gave informed written consent and were allocated to focus groups of three to five people. Workshop resources were recirculated to participants 1 week prior to focus groups. The topic guide (Additional file 1) was co-designed by all four authors to explore motivations for engaging in SusQI learning and practice, and what educational value had been created including application of learning in the clinical context. We piloted the focus groups with two non-participating medical students who helped refine the facilitation guide. We used a semi-structured approach so that all topics were covered, but emergent areas of interest explored as they arose. OM and KLG debriefed after each focus group, and specific areas of interest (including challenges to getting started on SusQI projects) were explored in greater depth in each subsequent focus group. Each lasted approximately 1 hour and was transcribed by OM assisted by automated software (Otter.ai) adding speaker identifiers and verbal cues (emphatics, pauses, laughter). Finally, transcriptions were proofread by KLG against the original recordings so that both authors were fully immersed in the data.

### Data analysis

**Fig. 1:**
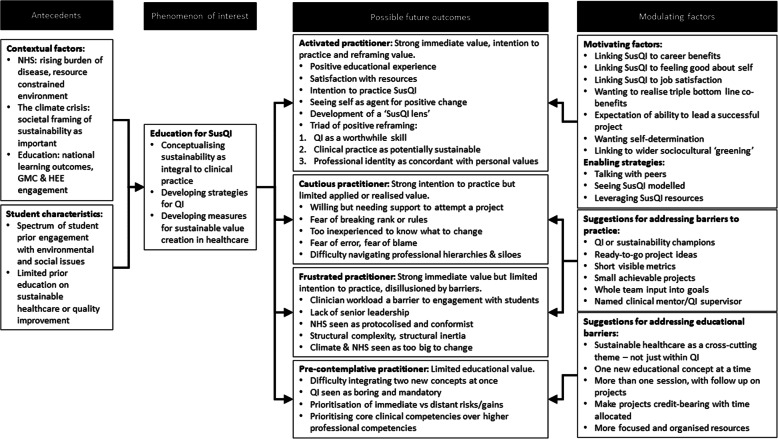
**Translating SusQI education into practice: themes from focus groups arranged into a categorical framework.** This framework was based on a qualitative analysis of focus groups involving third year medical students at Bristol Medical School who had undertaken Sustainability in Quality Improvement (SusQI) training

Our thematic analysis [16] began by coding core ideas line-by-line facilitated by NVivo 12 software. Core ideas were refined, merged where appropriate, audited against the underlying data, and grouped into themes through a process of consensual discussion. We used established theories to devise themes and prescriptive sub-themes relating to academic motivation and educational value [17]. Cook et al.’s integrative article suggests academic motivations can be a product of ‘goal orientation;’ ‘self-determination;’ the attribution of ‘potential benefits’ (attribution theory); the expectation of success (expectancy-value theory); or the social expectations of others (social-cognitive theory) [17]. Wenger-Traynor’s model suggests that educational value can be categorised as ‘immediate value’, ‘potential value’, ‘applied value’, ‘realised value’ or ‘reframing value’ [18] which relate to whether a participant found the learning engaging; whether they learned anything they considered useful; whether they applied what they learned; whether applying that learning created value for services/patients; or whether the learning fundamentally reframed their thinking. Other sub-themes were created inductively by grouping similar codes together. We continued to collect data until all new codes fitted into pre-existing themes, and we had rich data across the range of participants’ motivations, experiences, and self-reported outcomes. This was achieved by the final focus group. Themes were arranged into a categorical framework (Fig. 1) that explored differences between participants and the underlying factors that modulated those differences. Themes relating to the pandemic and teaching model are explored in our accompanying evaluation [13] and not reported here.

## Results

### How students conceptualised SusQI

Students tended to conceptualise sustainable healthcare in terms of resource usage such as reducing plastics or energy waste. The workshop also appeared to introduce more clinical conceptions. Participants talked about reducing over-investigation and over-treatment, reducing antibiotic misuse, choosing less damaging anaesthetic and inhaler options, reducing wasted labour through more effective and efficient clinical practices, and reducing the need for services through patient activation and disease prevention.

### Perceived educational value

There was strong student engagement with and appreciation of the workshop. Students described learning skills that they wanted to use as well as positively reframing their thinking about quality improvement, healthcare, and their future professional role. Very few, however, had attempted SusQI projects, and most reported multiple barriers to applying their learning or generating sustainable value for services. Of the 17 students who participated, only two had started SusQI projects.

### Who did it work for and why?

We were able to identify four distinct categories of talk:

1. students who fully engaged and pro-actively sought opportunities to apply their learning in the clinical context;
2. students who expressed internal barriers such as feeling too junior or lacking the confidence or capability to act;
3. students who expressed external barriers such as frustration at the structural rigidity of the healthcare system or the achievability of change;
4. students who did not want to engage as they saw sustainability and QI as outside their professional role or interest.

We have used these to suggest four potential outcomes of teaching, presented in Fig. 1. These are discussed below in relation to preceding factors, motivations, and other modulating factors. Quotes are identified according to focus group letter (A-E) and participant number. Square brackets and ellipses indicate where verbatim quotes have been clarified or condensed.

### Outcome 1: the activated SusQI practitioner

The SusQI workshop appeared most successful in activating students who had already prioritised sustainability. These participants recognised a spectrum of sustainability and identified themselves as more sustainably minded than most. They already valued and were familiar with concepts of sustainability, however, the workshop reframed their thinking about healthcare as a contributor to the global health emergency and ecological degradation.

External motivators such as career benefits, mitigating future threats, avoiding guilt, and associated prestige were common activating factors across all student groups, however these sustainably minded students also expressed intrinsic motivations and values such as curiosity, altruism, and self-determination. We found no comments explained by goal-orientation, perhaps because their SusQI projects were not summatively assessed. Activated practitioners expressed a set of core beliefs: sustainability action as necessary, the healthcare system as currently unsustainable, SusQI as a helpful tool, change as possible, the workplace as welcoming of improvement, and action as a professional duty.

Workshop content demonstrating the scale of unsustainable practices in healthcare troubled these students who felt alarmed that their involvement with the health service was out of step with their sustainable lifestyles and values.

*I think it just really took me aback, like ‘wow, [the health service plays] such a big role in [environmental destruction.]’ I always think that I'm quite a sustainable person generally, but I think I realise my involvement with the NHS [counteracts that], and that... I need to do something to balance out [my footprint]. D2*

They were also shaken by the impacts of environmental degradation on patients and healthcare: “*It made me realise that it’s going to directly impact my future career. And that was quite scary*” (A1).

Near-peer examples of successful projects enabled these students to reframe sustainability as within their control and influence, helping to mitigate those concerns. SusQI then became powerful tool for change through relatable examples of the SusQI framework which “put [QI] in a context that made us *want* to do it” (E1).

*I think it kind of made me more curious because it made me feel like I could make a change… if you’ve got the right research and you go about it the right way with the right process, you can make small changes that can make a difference. So, I think that was quite empowering and it kind of changed my impression of QI. C2.*

Interactive discussions during the workshop were valued, empowering students to identify and reflect on unsustainable workplace practices with peers. They expressed frustration that solutions were not yet in place but felt reassured by a like-minded peer community and the potential for change *“that actually felt like we could make a difference if we actually put some effort into this.”* (C3).

Participants were inspired by role models who framed SusQI as *“part of the bubble of patient care”* (A2), whilst depicting the NHS as *“an ever-evolving system”* (A3), which would ultimately embrace sustainability.

*[This workshop showed me] that people are willing to change like, I never really realised that the NHS is supportive of these changes, like they want to help decrease their environmental emissions and things because there are so many benefits… actually, it's been quite optimistic. A1*

Activated students worked to overcome barriers, for example, leveraging workshop resources to mitigate hierarchy and broach conversations about change.

*Almost like a slip of paper to wave at your colleagues saying "Look, this is why we have to [make sustainable change] It's not just my own personal, you know, want" or anything. Sorry! B4*

They conceptualised sustainability as an integral part of their future clinical practice rather than a separate siloed concept:

*It's made me more aware in my understanding that everything's interlinked… "patient care, my own health," you know, "education, treatment," it's all interlinked with sustainability rather than sustainability being this separate thing. C1*

The workshop transformed the way these students saw previously accepted practices, developing a new critical sustainability lens or ‘SusQI lens' which prompted them to start up conversations with clinicians.

*… rather than just following things for no reason, kind of looking for improvements and going in with that kind of "QI head on your shoulders" C2*

*When I do go back to the hospital, it will be [a case of] trying to start up conversations with people more. So, if you notice, like, "oh, like, that's a lot of waste." A1*

The SusQI workshop had a strongly positive impact on their professional identity, creating “*a sense of meaning within your job, even greater than just [the job] itself*” (D1). The workshop was also successful in creating a sense of leadership and agency *“that actually, we have the capability to make a massive difference... quite inspiring... you wanted to go out and help”*“ (A1)*.* Another noted “*it was something that I anecdotally told other people about because it impacted on me so much*” (E1).

**Fig. 2:**
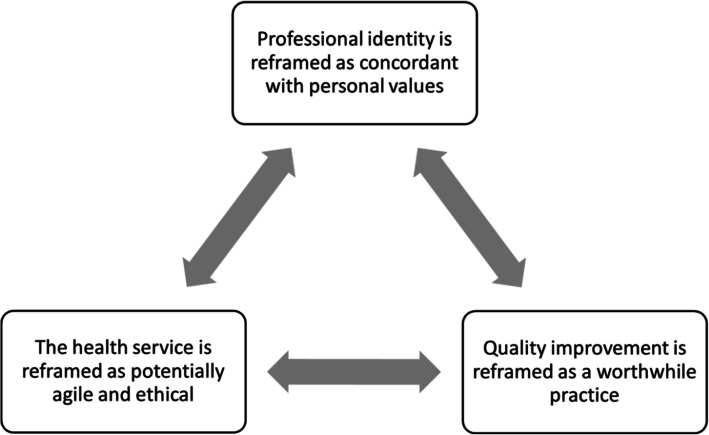
**A triad of positive reframing related to SusQI teaching.** This analysis was based on focus groups involving third year medical students at Bristol Medical School who had undertaken Sustainability in Quality Improvement (SusQI) training

We identified a triad of positive reframing in this group, illustrated in Fig. 2. Activated students newly identified themselves as agents for positive change, their profession as potentially ethical and sustainable, and QI as a worthwhile practice.

### Outcome 2: the willing but cautious SusQI practitioner

Willing but cautious participants saw sustainable healthcare as important and potentially achievable, however they expressed personal and interpersonal barriers to action. They described expectations to conform when shadowing on placement, which included adopting seniors’ unsustainable practices. Tacit professional boundaries prevented these students suggesting new or improved practices.

*I don't know how the wards run… Managerial staff will know more than I do… And so yeah… Going onto the ward and trying to bring about changes is terrifying. A2*

They explained that students *“don’t feel comfortable enough to be able to broach that conversation to a consultant or anyone else”* (C4). They felt it would be inappropriate to educate their busy seniors. These students identified themselves at “*the bottom of the food chain”* (A2) in a hierarchical organisation, lacking the social capital or knowledge to push for sustainability. Constantly rotating between specialties and hospitals left them without insights into local expertise, systems, or processes.

*To suddenly come up and say, “Can we look at [sustainability as well]”, it might seem slightly [brash, over-confident] … I don’t feel in the place [of authority] where I can do that right now.* (C3).

In contrast, they perceived senior doctors as possessing the influence to drive SusQI projects but unapproachable and lacking awareness or time, undermining the achievability of SusQI.

*I think educating more senior doctors [is needed]. Once more senior positions start doing something you think “I could do that” and make it more likely for people to listen and do something about it.* (C1)

**Fig. 3:**
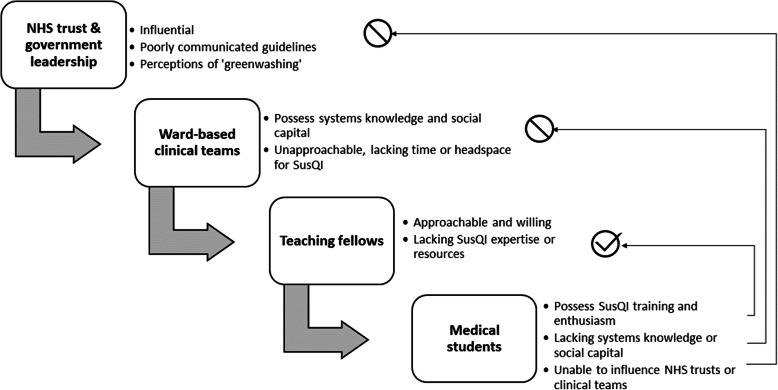
**Perceptions of the clinical hierarchy and medical students’ sphere of influence in relation to SusQI.** Arrows indicate medical students’ perceived influence within the clinical hierarchy. This analysis was based on focus groups involving third year medical students at Bristol Medical School who had undertaken Sustainability in Quality Improvement (SusQI) training

Participants described a range of interpersonal challenges across the clinical hierarchy relating to mismatches between intention to act, power to act, capacity to act and SusQI knowledge. Our results in relation to the clinical hierarchy are summarised in Fig. 3. Participants suggested appointing established team members as sustainability champions to collate and advocate for SusQI ideas and to act as role models.

*If they'd previously put their name out there as someone who would be interested in something like this… then yeah, I would be more inclined to go forward [and approach them].* (C3)

*I feel quite daunted by the idea of having to go and speak to doctors myself and bring about a change myself. Doesn't necessarily have to be doctors, it could be nurses, it could be [healthcare assistants], could be anyone [in the healthcare team] really, couldn't it?* (A2)

### Outcome 3: the frustrated SusQI practitioner

Students in this group valued and were motivated by the SusQI workshop, however, barriers relating to their junior status were compounded by perceptions of structural barriers in the clinical workplace. These combined to undermine their belief in the achievability of SusQI, leading to disengagement and frustration.

*I've sent numerous emails… It's like [I get] blocked, people don’t… have anything for me to do… I feel quite disheartened that [QI] was this big, like, exciting thing you can get involved in and it's actually, in reality, it's not… The actual getting [a project] is really difficult.* (E1)

They also described a disconnect between senior and junior engagement and criticised the absence of sustainability guidance from the NHS or University *“if everyone’s been told the same thing, you’re working together to do it”* (B2). Clinical administration was described as over-regulated, complex, and mysterious, while clinical teams were busy and resistant to change. The hectic and often stressful healthcare workplace was described as lacking the psychological space to engage clinical staff in SusQI planning.

*Trying to find the time and headspace to stand and, just thinking ‘how [we] could change practice for sustainable benefit?’ That's quite difficult.* (E1)

Where sustainability guidelines were in place, contradictory actions within a local NHS trust epitomised the greenwashing perceived by students, who expressed disillusionment that they were not being followed. They felt that large-scale sustainability appeared unrealistic *“until cultural change happens at the lower levels”* (B1). Conformity to clinical guidelines was described as part of patient safety but also a safety blanket for clinical staff, with change perceived as disruptive. Systems were considered too complex to adapt and fear of blame stifled innovation. Frustrated participants expressed a dilemma: whether to persevere with action, or to stick with current practices and accept the associated moral injury:

*The ethical implications of not doing things sustainably are quite high for future generations... It’s a big ethical problem. (D2)*

*Like, putting yourself in the firing line [if you question it]. But it would be so much easier just to keep on going, and using all this PPE, and throwing it in the bin, and going home and [getting praised] "you've done such a great job [on the COVID front-line]." But in reality, you're acting incredibly unsustainably. (E4)*

### Outcome 4: the pre-contemplative SusQI practitioner

Students in this group were not ready to engage with SusQI expressing a variety of different reasons. They described sustainable healthcare concepts as unfamiliar: *“[It was] the first time.... that we’ve talked [in medicine] about environmental issues… [we] would obviously really love to integrate it, but... [we] need more information and about how to do that”* (E3). These students suggested that sustainable healthcare needed to be an integral theme, rather than confined to QI teaching.

*But I feel like one lecture will not just change how I act and how I - sort of - work. I feel like it needs to be a continuous thing of building upon what we've learnt already… you're not going to learn one thing from one lecture and change the entire way you act, you need to keep on sort of building up on that, I think.” (A2)*

Unsustainable healthcare and ecological breakdown were perceived as an insurmountable far away problem that someone else would sort out: “*... I think a lot of people feel like it’s not actually helping themselves. So, it’s easier... to leave it to other people to deal with it”* (B2). They prioritised the health of patients today over the health of wider populations tomorrow, despite acknowledging the exponential consequences for future generations.

*What you do now doesn't have any immediate ramifications for you but has… everlasting ramifications for ‘this many’ people. But since it doesn't affect you, you don't pay attention to it.* (B3)

QI projects had a prior reputation as “*boring... something that you had to do*” (D2), and preventative health was described as too abstract and less exciting than patient-facing acute medicine.

*If you're [undertaking QI] there's not that immediate kind of satisfaction of "I can do this"… it's the cliche thing to say, I've gone into medicine because I want to help people... There's more immediate satisfaction [in helping patients] than there would be with QI projects, I think.* (C1)

*I'm not gonna lie. I think when I hear quality improvement, it just … kind of just, like makes me start snoring a little bit.* (B1)

Pushing for change was seen as exhausting and beyond their professional scope.

*Yeah, it gets a bit exhausting if you're thinking about how to improve every aspect of your job at all times.* (B1)

Motivation to persevere was related to expected success rate, with students dissuaded by rumours that “*QI failing is brutal”* (C1). For these students, altruistic motivations did not appear to justify the perceived effort and risks of engagement.

**Fig. 4:**
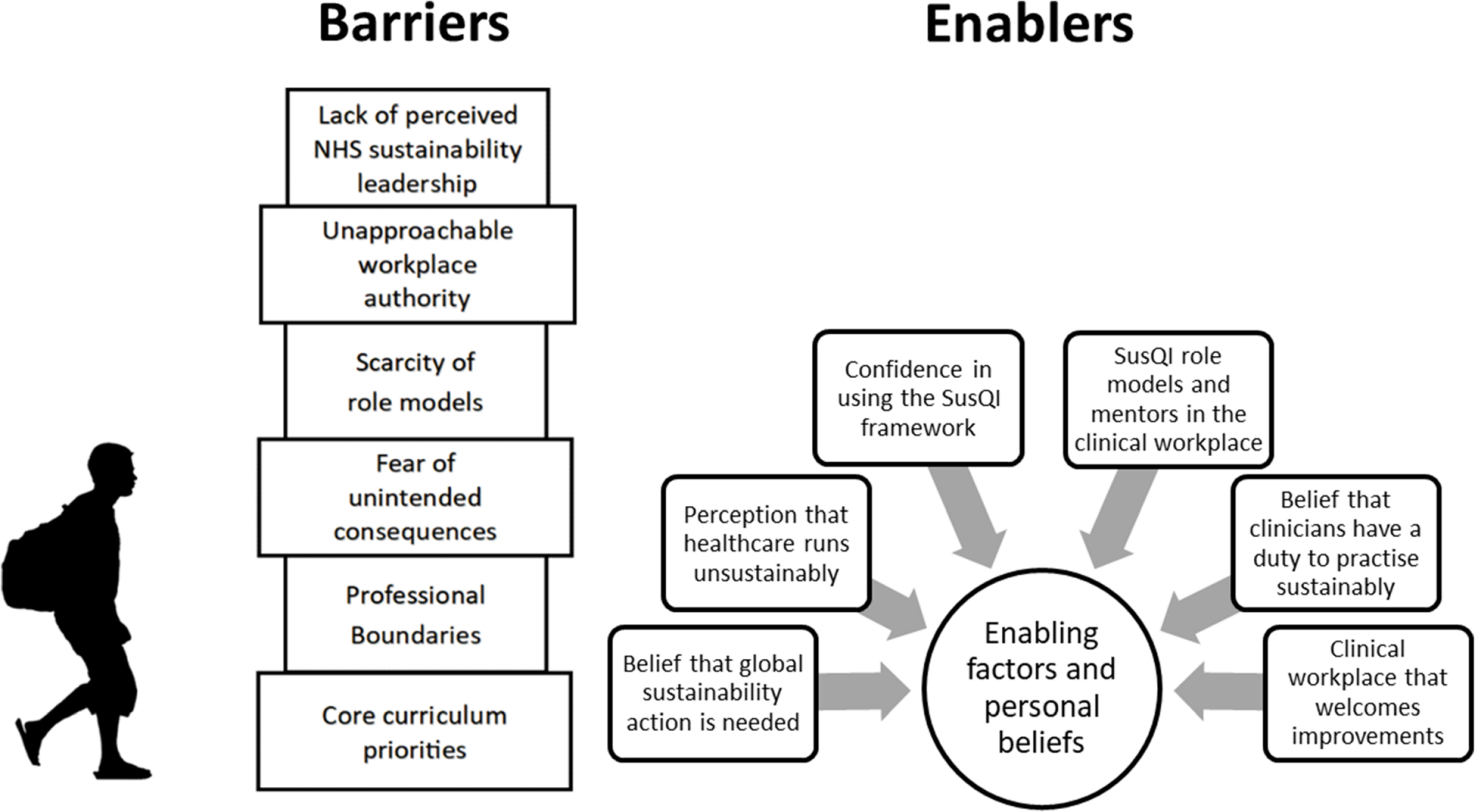
**A summary of barriers and enablers to applying SusQI to the clinical context.** This analysis was based on focus groups involving third year medical students at Bristol Medical School who had undertaken Sustainability in Quality Improvement (SusQI) training

An integrated summary of results relating to barriers and enabling factors across all outcome groups is depicted in Fig. 4.

## Discussion

We have presented an analysis of five focus groups involving 17 third year medical students exploring self-reported academic motivations and experiences relating to SusQI education as well as perceptions of the barriers to and enablers of translating workshop learning to the clinical context (Fig. 4). We have identified four potential outcomes of this teaching: activated, cautious, frustrated and pre-contemplative SusQI practitioners. Activated students tended to be already sustainably minded, internally, and altruistically motivated, and experienced a triad of positive reframing: linking their personal values to their future professional practice through QI practices. Cautious students felt too junior to suggest change, however, they expressed willingness to engage with support and permission. Frustrated students saw both the sustainability of the NHS and environmental degradation as intractable problems and were discouraged by perceived infrastructural barriers, with concomitant moral injury. Pre-contemplative students expressed a variety of reasons for not engaging, including seeing sustainable healthcare practices as unfamiliar or beyond their professional scope and QI as too difficult or beyond their clinical interests, preferring nearer more tangible rewards.

We discuss these findings in relation to Michie’s theoretical domains framework [19] which describes how new behaviours or actions are not a merely behavioural pre-disposition, but rather a product of capability, opportunity, and motivation with associated domains of evidence-based strategies for change [20]. Activated learners clearly felt motivated and capable of leading change, suggesting that opportunity was their principal need. Cautious and frustrated learners expressed needs relating to psychological or physical capability – feeling too junior or that change was not achievable. Pre-contemplative learners’ needs related principally to motivation – feeling uninterested in QI or that sustainability was not part of their future role. These needs were cumulative, so once motivated, students needed to feel that they had the right skills and could make a positive difference, and finally all students needed the opportunity to act.

**Fig. 5:**
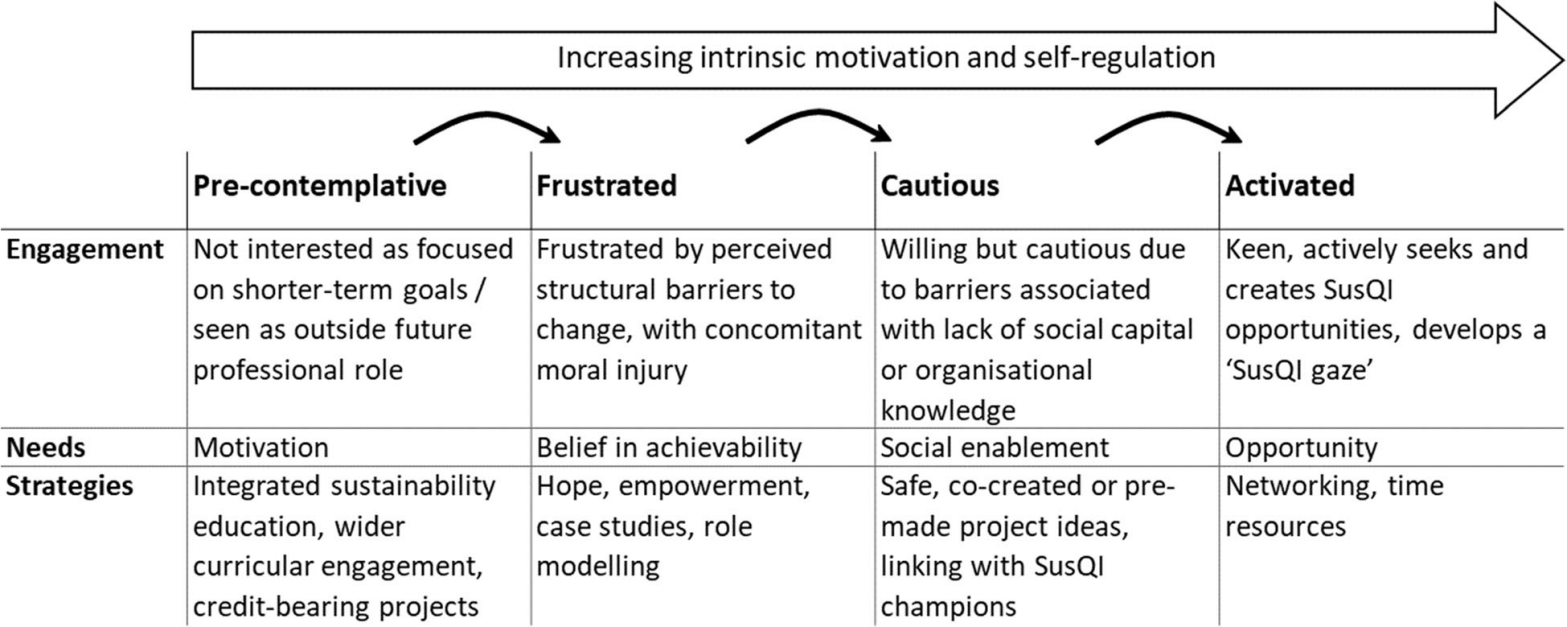
Conceptual model for achieving the necessary conditions for action. This analysis was based on focus groups involving third year medical students at Bristol Medical School who had undertaken Sustainability in Quality Improvement (SusQI) training. It was informed by theories of motivation, educational outcome, and behaviour

Figure 5 presents these four potential outcomes on a spectrum of activation and self-regulation with our analysis of these students’ needs and potential strategies for enabling action.

Applying the intervention functions from Michie’s theoretical domains framework to this spectrum of student needs enables us to make theoretically informed recommendations for educators. Michie’s framework suggests pre-contemplative students could be supported through incentivisation and education. This could be achieved through alignment of project outcomes to students’ current goals, or through reframing their social and professional identity to incorporate longer term goals. Such strategies might include credit-bearing projects, integrating sustainability education more consistently throughout their medical degree and postgraduate training, and including personally congruent role models in SusQI workshops to positively influence their professional identity. Frustrated students could be supported by building belief in their capabilities and optimism through seeing near-peers model achievable but impactful projects, and having structural inertia addressed by introducing sustainability into wider multi-professional QI training. Cautious students could be supported through enablement strategies and social support, for example project ideas that were co-created with clinical teams, so that students felt they were working within acceptable norms and not breaking unspoken rules, with SusQI champions to provide ongoing social permission, support, and encouragement. This is supported by previous research that suggests that student projects are more often fruitful when linked to hospital agendas and where there is organisational momentum [21]. Finally, activated students could be supported through modelling, enablement, and infrastructural changes, for example through access to QI networks, resources, and allocated time for projects.

The national organisations promoting the SusQI toolkit, including Health Education England and the Centre for Sustainable Healthcare, are working to rapidly propagate concepts of sustainability within postgraduate and wider health professional QI teaching. This should make it progressively easier for students to get involved if clinical teams are already engaged and familiar with the framework.

We acknowledge that SusQI is a complex field which may not appeal to every clinician, and that some of the technical aspects of achieving sustainable healthcare may require specialised implementation scientists. However, sustainable clinical practice and QI skills are now compulsory undergraduate learning outcomes in the UK [5] and a crucial contributor to NHS England’s goal of carbon neutrality by 2040 [4]. We suggest that further implementations of the SusQI toolkit move beyond evaluating what was taught or learned and focus on translation of learning into tangible benefits to services.

### Strengths and limitations

This research has built theory from an exploration of a local phenomenon based on one workshop with associated resources and as such, it should be seen as programme-level theory, rather than mid-range theory or grand theory [20]. Our findings therefore need to be interpreted within this context and translation to other healthcare schools should be based on local knowledge. We used convenience sampling for focus groups and participants may not be representative of the student population. Our population was limited to third-year medical students; therefore, further research to investigate whether students change their perception of SusQI over the course of their medical education is needed.

## Conclusions

The workshop and resources were successful in building self-reported motivation and skills, as well as reframing learners’ perceptions of sustainability and quality improvement (Clery et al. 2021). Our findings highlight that ‘motivation to act’ and ‘knowing how to act’ may not be enough. Whilst some students can get started on SusQI projects, others feel limited by the social or cultural contexts in which they operate, and the wider physical and operational environments in which their practice exists. As junior members of the healthcare team, they may require additional support to apply their learning and realise sustainable value for services. We have suggested several theoretically informed recommendations for SusQI educators. These include wider curricular engagement with concepts of sustainable clinical practice; supportive workplace enablement strategies such as incorporation of sustainability into existing postgraduate QI training, workplace champions, and co-creation of improvement goals; and finally, ensuring time and headspace for students to engage through opportunities for credit-bearing project work. Further research is needed to explore the impacts of these recommendations.

## Supplementary Information

**Additional file 1.**

## Data Availability

The datasets generated and/or analysed during the current study are not publicly available as this was not included within our consent, but are available from the corresponding author on reasonable request.

## Abbreviations

SusQI: Sustainability in Quality Improvement
QI: Quality Improvement
GMC: General Medical Council
NHS: National Health Service

## References

[1] Costello A, Abbas M, Allen A, Ball S, Bell S, Bellamy R (2009). Managing the health effects of climate change: lancet and University College London Institute for Global Health Commission. Lancet.

[2] Wang H, Horton R (2015). Tackling climate change: the greatest opportunity for global health. Lancet.

[3] Sustainable Development Unit (2018). What is sustainable health?.

[4] NHS England and NHS Improvement (2020). Delivering a ‘net zero’ National Health Service.

[5] GMC (2018). Outcomes for Graduates.

[6] Health Education England (2020). The Future Doctor Programme: A co-created vision for the future clinical team.

[7] Mortimer F, Isherwood J, Pearce M, Kenward C, Vaux E (2018). Sustainability in quality improvement: measuring impact. Future Hosp J.

[8] Mortimer F, Isherwood J, Wilkinson A, Vaux E (2018). Sustainability in quality improvement: redefining value. Future Hosp J.

[9] Mortimer F (2010). The sustainable physician. Clin Med.

[10] Centre for Sustainable Healthcare (2020). Sustainability in Quality Improvement.

[11] Dixon T, Colantonio A, Ganser R, Carpenter J, Ng’ombe A (2009). Measuring socially sustainable urban regeneration in Europe.

[12] Smith AC, Holland M, Korkeala O, Warmington J, Forster D, ApSimon H (2016). Health and environmental co-benefits and conflicts of actions to meet UK carbon targets. Clim Pol.

[13] Clery P, D'Arch Smith S, Marsden O, Leedham-Green K (2021). Sustainability in quality improvement (SusQI): a case-study in undergraduate medical education. Pre-publication communication.

[14] Heron J, Reason P (1997). A participatory inquiry paradigm. Qual Inq.

[15] Djohari N, Higham R (2020). Peer-led focus groups as ‘dialogic spaces’ for exploring young people’s evolving values. Camb J Educ.

[16] Braun V, Clarke V (2012). Thematic analysis. APA handbook of research methods in psychology, Vol 2: Research designs: Quantitative, qualitative, neuropsychological, and biological. APA handbooks in psychology®.

[17] Cook DA, Artino AR (2016). Motivation to learn: an overview of contemporary theories. Med Educ.

[18] Wenger E, Trayner B, De Laat M (2011). Promoting and assessing value creation in communities and networks: a conceptual framework.

[19] Cane J, O’Connor D, Michie S (2012). Validation of the theoretical domains framework for use in behaviour change and implementation research. Implement Sci.

[20] Michie S, van Stralen MM, West R (2011). The behaviour change wheel: a new method for characterising and designing behaviour change interventions. Implement Sci.

[21] Wong BM, Etchells EE, Kuper A, Levinson W, Shojania KG (2010). Teaching quality improvement and patient safety to trainees: a systematic review. Acad Med.

