# Supplementary material for: Sustainability in Quality Improvement (SusQI): challenges and strategies for translating undergraduate learning into clinical practice
Source: BMC Med Educ. 2021 Oct 30;21:555. doi: 10.1186/s12909-021-02963-7 (PMC8556782; doi:10.1186/s12909-021-02963-7)
Supplement: Supplementary file 1 — Additional file 1. [file 12909_2021_2963_MOESM1_ESM.docx]

**Appendix 1: Topic Guide**

| **Section** | **Topic initiation question**   - *Prompts (as needed)* |
| --- | --- |
| **Warm up** | "Talk to each other, not me. There are no wrong answers!"  "I'm also interested if you disagree with each other, so please add on to one another!"   - *Simple, short ground rules* - *Allow time for participants to introduce to each other* |
| 1: scene setting, subject recap | **What do you understand Quality Improvement (QI) to be?**   - *How is this relevant to health care?* - *How is this relevant to day-to-day practice as a doctor?*   **What do you understand about sustainability in healthcare?**   - *What does it mean to be 'sustainable'?* - *What does it mean for a hospital, ward, or medication to be 'sustainable'?* - *What do you understand about the "triple bottom line" concept? (Economic, Social & Environmental impacts)* |
| **Main section** |  |
| 2: immediate value | **What parts of the workshop highlighted the link between sustainability & QI?**   - *What did you think about linking Sustainability to QI?* - *How did you feel the workshop was delivered?* - *How did it fit in with other workshops? (Iv)* - *What were the most meaningful parts? (Iv)* |
| 3: applied / realised value | **Have you applied knowledge from this workshop?**   - *Did this workshop impact your QI project plan or outcome?* - *How did the SusQI workshop contribute?* - *If not, why not?* |
| 4: intentional value | **Would you change your clinical practice day-to-day, or in the future? In what way?**   - *Are you interested in future (sustainable) QI initiatives?* - *Would you consider making sustainability a target outcome in future QI projects?* |
| 4: reframing value | **Has this workshop reframed your perspective on anything in any way?**   - *Did linking QI with sustainability change:* - *Your perception of what QI is?* - *Your perception of sustainability?* - *Perception of your role as a doctor in the NHS?* - *Your motivation to undertake QI?* - *Belief in your ability to make a successful QI project?* - *Your curiosity about QI?* - *Any other impacts on you?* - *Your lifestyle?* - *Your motivation to act on sustainability?* - *Your concerns about sustainability?* |
| **Closing section** | Ask group to rephrase any ambiguous/surprising answers   - *Anything you would like to add?*   Thanks, questions, and wind down. |
